# Identification and Pharmacological Characterization of Two Serotonin Type 7 Receptor Isoforms from *Mythimna separata*

**DOI:** 10.3390/ijms24010655

**Published:** 2022-12-30

**Authors:** Wenbo Chen, Xiaoyan Gao, Huixin Wang, Guiying Xie, Shiheng An, Yongkun Du, Xincheng Zhao

**Affiliations:** 1 Henan International Joint Laboratory of Green Pest Control, College of Plant Protection, Henan Agricultural University, Zhengzhou 450046, China; 2 International Joint Research Center of National Animal Immunology, College of Veterinary Medicine, Henan Agricultural University, Zhengzhou 450046, China

**Keywords:** *Mythimna separata*, serotonin, 5-hydroxytryptamine receptor 7, alternative splicing, pharmacology, expression profiles

## Abstract

Serotonin (5-hydroxytryptamine, 5-HT) is an important neuroactive molecule, as neurotransmitters regulate various biological functions in vertebrates and invertebrates by binding and activating specific 5-HT receptors. The pharmacology and tissue distribution of 5-HT receptors have been investigated in several model insects, and these receptors are recognized as potential insecticide targets. However, little is known about the pharmacological characterization of the 5-HT receptors in important agricultural pests. In this study, we investigated the sequence, pharmacology, and tissue distribution of *5-HT7* receptors from oriental armyworm *Mythimna separata* (Walker) (Lepidoptera: Noctuidae), an important migratory and polyphagous pest species. We found that the *5-HT7* receptor gene encodes two molecularly distinct transcripts, *Msep5-HT7L* and *Msep5-HT7S*, by the mechanism of alternative splicing in *M. separata*. Msep5-HT7S differs from Msep5-HT7L based on the deletion of 95 amino acids within the third intracellular loop. Two Msep5-HT7 receptor isoforms were activated by 5-HT and synthetic agonists α-methylserotonin, 8-hydroxy-DPAT, and 5-methoxytryptamine, resulting in increased intracellular cAMP levels in a dose-dependent manner, although these agonists showed much poorer potency and efficacy than 5-HT. The maximum efficacy of 5-HT compared to the two 5-HT isoforms was equivalent, but 5-HT exhibited 2.63-fold higher potency against the Msep5-HT7S than the Msep5-HT7L receptor. These two isoforms were also blocked by the non-selective antagonist methiothepin and the selective antagonists WAY-100635, ketanserin, SB-258719, and SB-269970. Moreover, two distinct mRNA transcripts were expressed preferentially in the brain and chemosensory organs of *M. separata* adults, as determined by qPCR assay. This study is the first comprehensive characterization of two splicing isoforms of 5-HT7 receptors in *M. separata*, and the first to demonstrate that alternative splicing is also the mechanism for producing multiple 5-HT7 isoforms in insects. Pharmacological and gene expression profiles offer important information that could facilitate further exploration of their function in the central nervous system and peripheral chemosensory organs, and may even contribute to the development of new selective pesticides.

## 1. Introduction 

Serotonin (5-hydroxytryptamine, 5-HT) is an important biogenic amine, as the neurotransmitter/neuromodulator participates in various critical physiological functions in both vertebrates and invertebrates [1,2,3,4]. For instance, serotonin plays a role in many vital human physiological processes, such as aggression, emotion, sleep, appetite, sexual activity, and learning ability [1,3]. Concerning insects, which are invertebrates, several studies have shown that 5-HT regulates many comparable physiological processes, such as aggression, sleep, and circadian rhythm, in *Drosophila melanogaster* Meigen (Diptera: Drosophilidae) and *Gryllus bimaculatus* De Geer (Orthoptera: Gryllidae) [5,6,7,8]; feeding, learning, and memory in *D. melanogaster* and *Apis mellifera* Linnaeus (Hymenoptera: Apidae) [9,10,11,12,13]; and olfactory responses in *D. melanogaster* and *Manduca sexta* (Linnaeus) (Lepidoptera: Sphingidae) [14,15,16]. 

In vertebrates, the effects of serotonin are mediated by its binding to two specific types of receptors: ionotropic and metabotropic receptors. All metabotropic receptors belong to G protein-coupled receptors (GPCRs), with seven transmembrane domains that are divided into six distinct classes based on amino acid sequence homology, pharmacological properties, and downstream second messenger pathways (5-HT1, 2, 4, 5, 6, 7), while the remaining 5-HT3 receptor is an ionotropic receptor [1,2,3]. In most insects, three classes of 5-HT receptors have been found (5-HT1, 5-HT2, and 5-HT7 type receptors), based on sequence similarities and activated second-messenger pathways with their counterparts in vertebrates [3,4,17,18]. Within the same receptor class, the signaling properties seem to be very well maintained between insects and vertebrates [3]. The 5-HT1 type receptors couple preferentially to G_i/o_ proteins, and thus inhibit cyclic adenosine monophosphate (cAMP) synthesis [4,17]. The 5-HT2 receptors couple to G_q/11_ proteins, causing an increase in cytosolic Ca^2+^ concentration [12,19], while 5-HT7 receptors couple to G_s_ proteins, activating adenylyl cyclase (AC) activity and hence increasing intracellular cAMP formation [3,4]. Moreover, Qi et al. [20] recognized a novel 5-HT8 receptor in *Pieris rapae* (Linnaeus) (Lepidoptera: Pieridae) that mediated increased intracellular calcium concentration, which may be an invertebrate-specific receptor without a counterpart in mammals. The pharmacological characteristics of the same type of 5-HT receptors vary significantly among different insect species [3,21]; therefore, 5-HT receptors are recognized as potential insecticide targets [3,4]. In fact, Cai et al. [22] demonstrated that the 5-HT1A receptor agonist PAPP and its derivatives in *Haemonchus contortus* (Rudolphi) Cobb (Rhabditida: Trichostrongylidae) could inhibit the growth or kill the larvae of *Mythimna separata* (Walker) (Lepidoptera: Noctuidae).

The *5-HT7* receptor genes have been cloned in many vertebrates. In humans, mice, and rats, the coding region of this gene contains three introns, and alternative splicing at the second and third introns is a general mechanism that results in a number of splice variants. No major differences in their pharmacology or functionality have been reported so far [23], and all 5-HT7 receptor isoforms are coupled to G_s_ proteins and stimulate cAMP formation [2]. On the contrary, only one *5-HT7* receptor gene transcript has been cloned in insects thus far [3]. Tissue distribution and functional studies have confirmed that 5-HT7 plays a vital role in circadian rhythm, rapid eye movement sleep, thermoregulation, depression, anxiety, schizophrenia, nociception, epilepsy, and memory learning in mammals [23,24,25]. The 5-HT7 receptor has been characterized in several model insects, and has been shown to be involved in normal courtship and mating behaviors in *D. melanogaster* [26], learning and memory in *A. mellifera* [27], and saliva secretion in *Calliphora vicina* Robineau–Desvoidy (Diptera: Calliphoridae) [28]. The pharmacological properties and physiological functions of the 5-HT7 receptor in important agricultural pests remain largely unknown.

In the present study, we cloned the *5-HT7* receptor gene from *M. separata*, one of the important polyphagous insect pests in China, costing more than ¥ 146 million ($18 million) in insecticide management on wheat alone in 1997 [29]. Different from *5-HT7* receptors gene transcription patterns in other insects [26,27,28], this gene encodes two molecularly distinct transcripts by the mechanism of alternative splicing in *M. separata*. To our knowledge, this is the first report showing that alternative splicing is the mechanism for producing multiple 5-HT7 isoforms in insects. Furthermore, we examined the pharmacological characteristics and expression profiles in different tissues of *M. separata* larvae and adults. These results provide a foundation for understanding the actions of serotonin in behavior and physiological function, and for exploring novel pesticides to control this pest in the future.

## 2. Results 

### 2.1. Molecular Features and Phylogenetic Analysis

**Figure 1:**
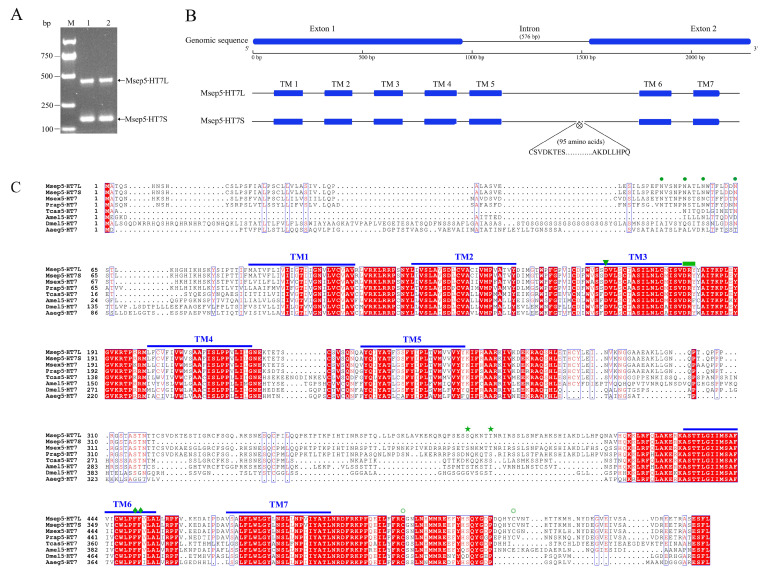
Amino acid sequence alignment of two Msep5-HT7 isoforms with representative homologous receptors. (**A**) Two molecular isoforms of *Msep5-HT7* receptor mRNA detected by PCR. Lanes 1 and 2 used cDNA template from adult brains and antennae of *M. separata*. (**B**) Schematic representation of the *Msep5-HT7* receptor genome and two molecular mRNA isoforms detected by PCR. ⊗ in the Msep5-HT7S schematic indicates deletion of 95 amino acids. Predicted transmembrane regions are indicated as TM1-TM7. (**C**) Sequences were aligned using MAFFT. Identical residues in all sequences are shown in white against a red background, and conserved substitutions are shown in red. Seven predicted transmembrane domains are indicated by blue bars above sequences (TM1-7). Dots (●) indicate putative N-glycosylation sites above sequences in the N-terminal region; stars (★) and concentric circles (○) indicate potential phosphorylation sites for protein kinase C and putative palmitoylation sites, respectively; triangles (▼) indicate aspartic acid residue predicted to be involved in agonist binding; diamonds (♦) indicate the pairs of phenylalanine after FxxxWxP motif in TM6, a unique feature of aminergic receptors; rectangles (▄) indicate the highly conserved DRY motif, which is believed to have a key role in receptor activation.

The full length of genomic DNA and cDNA of *Msep5-HT7* from adult *M. separata* was amplified by using transcriptome analysis with a PCR-based strategy. The *Msep5-HT7* gene contains an intron and produces two molecularly distinct transcripts (*Msep5-HT7L* and *Msep5-HT7S*) by the mechanism of alternative splicing (Figure 1A). The open reading frame of *Msep5-HT7L* comprises 1695 bp and codes for 564 amino acid residues, with a calculated molecular weight of 63.25 kDa. Msep5-HT7S differs from Msep5-HT7L based on the deleted of 95 amino acids within the third intracellular loop (Figure 1B). The Msep5-HT7 receptors were predicted to be GPCRs, with seven transmembrane domains (TM1–7) connected by intra- and extracellular loops (Figure 1C). Sequence motifs in 5-HT receptors that are essential for ligand binding and signal transduction are well conserved in two Msep5-HT7 isoforms, including the highly conserved D-R-Y motif at the N-terminus of the second intracellular loop, a conserved aspartic acid in TM3, the sequence F-X-X-XW-X-P followed by a pair of phenylalanine residues within TM6, and two cystein residues in the intracellular C-terminus which are putative palmitoylation sites. In addition, the Msep5-HT7S receptor has a shorter third intracellular loop, and lacks two consensus sites for phosphorylation by protein kinase C ([S/T-X-[R/K]) compared with the Msep5-HT7L receptor (Figure 1C). 

**Figure 2:**
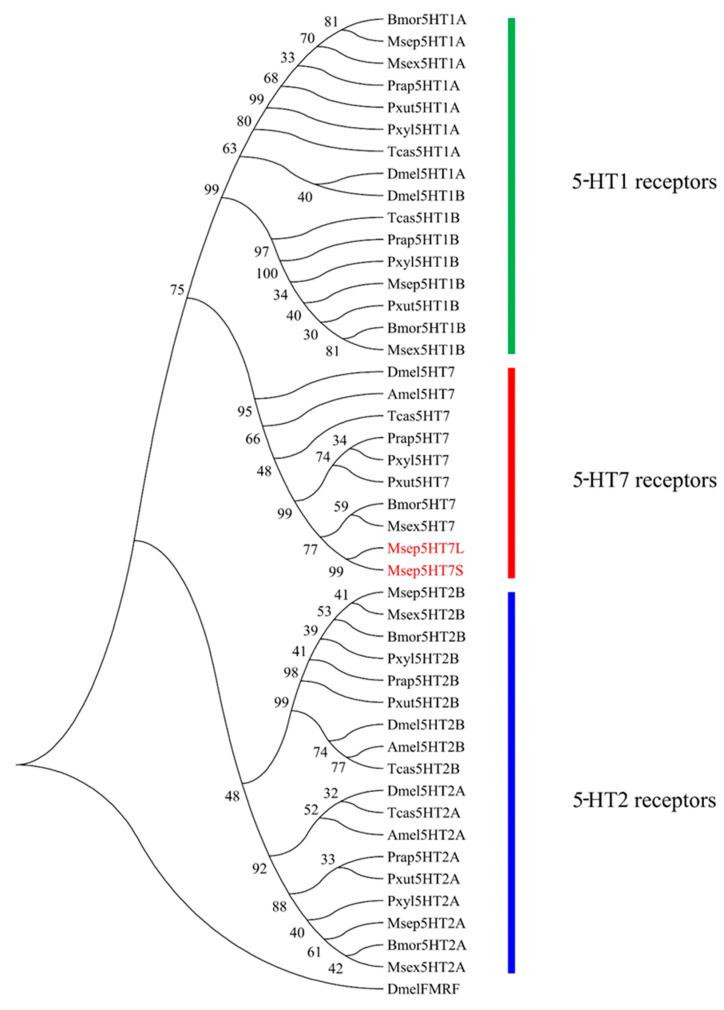
Phylogenetic analysis of two Msep5-HT7 receptor isoforms and various 5-HT receptors from other insects. Phylogenetic tree was constructed by MEGA6.0 with 1000-fold bootstrap re-sampling based on alignment results of MAFFT. FMRF amide receptor (DmelFMRF) of *Drosophila melanogaster* was used as outgroup. Bmor, *Bombyx mori*; Msep, *Mythimna separata*; Pxyl, *Plutella xylostella*; Prap, *Pieris rapae*; Pxut, *Papilio xuthus*; Msex, *Manduca sexta*; Dmel, *Drosophila melanogaster*; Amel, *Apis mellifera*; Tcas, *Tribolium castaneum*.

A comparison of the amino acid sequences of two Msep5-HT7 isoforms with other insect 5-HT7 receptor orthologues revealed high sequence similarities. The similarity between Msep5-HT7L and Msep5-HT7S is 83.2%. The similarity between Msep5-HT7L and lepidopteran insect 5-HT7 orthologues is 93.3% for *M. sexta*, 90.1% for *P. rapae*, 91.4% for *Bombyx mori* (Linnaeus) (Lepidoptera: Bombycidae), and 77.6% for *Papilio Xuthus* Linnaeus (Lepidoptera: Papilionidae). The similarity between Msep5-HT7S and lepidopteran insect 5-HT7 orthologues is 78.5% for *M. sexta*, 76.8% for *P. rapae*, 77.0% for *B. mori*, and 64.3% for *P. xuthus*. This clearly shows two Msep5-HT7 receptor isoforms clustered with other insect 5-HT7 receptors in a distinct clade, indicating that they belong to the 5-HT7 type receptors by phylogenetic tree analysis (Figure 2).

### 2.2. Pharmacological Characterization of Two Msep5-HT7 Receptor Isoforms

**Figure 3:**
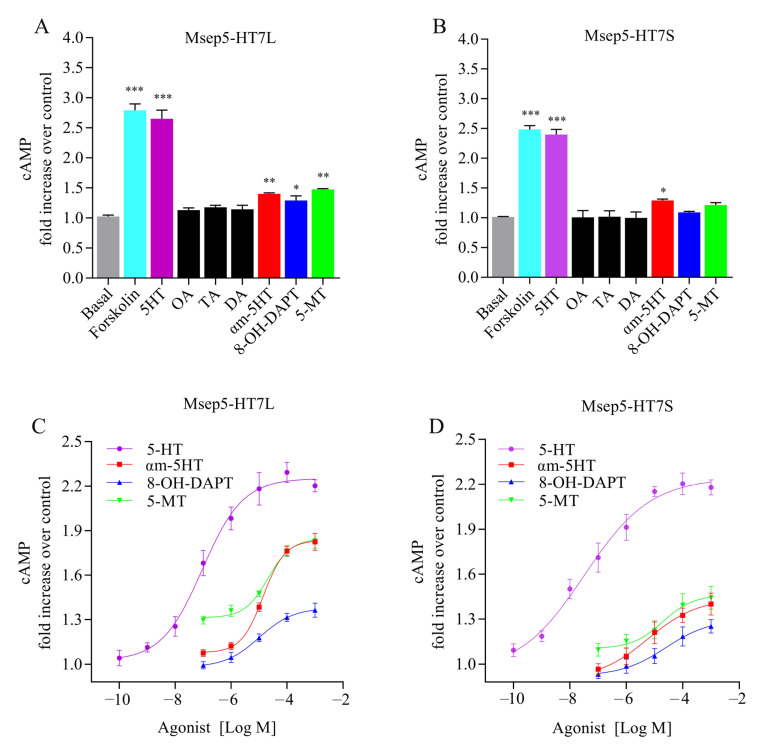
Effects of biogenic amines and agonists on intracellular cAMP levels in HEK293 cells expressing Msep5-HT7L (**A**,**C**) and Msep5-HT7S receptors (**B**,**D**). Results are presented as fold increase of cAMP levels relative to transfected cells treated with only DPBS buffer, defined as the basal level and normalized as 1. For (**A**,**B**), the concentration of biogenic amines and various synthetic agonists used was 10 μM, with 1 μM forskolin-treated transfected cells as a positive control. Dose-dependent effects of 5-HT, and several agonists on Msep5-HT7L and Msep5-HT7S receptors, are shown (**C**,**D**). Asterisks indicate values significantly different from basal values using one-way ANOVA followed by Dunnett’s multiple comparison test (* *p* < 0.05, ** *p* < 0.01, *** *p* < 0.001). Data are presented as means ± SD of three independent experiments.

In order to determine the ligand specificity of the two Msep5-HT7 receptor isoforms, we compared the ability of various biogenic amines (serotonin, octopamine, dopamine, and tyramine) and synthetic 5-HT receptor agonists (αm-5-HT, 5-MT, and 8-OH-DAPT) at a final concentration of 10 μM to alter intracellular cAMP levels in HEK293 cell lines that stably express either Msep5-HT7L or Msep5-HT7S receptors. Among the four tested biogenic amines, only 5-HT significantly increased cAMP levels in cells expressing either Msep5-HT7L or Msep5-HT7S receptors. All synthetic agonists activated the Msep5-HT7L receptor, while only αm-5-HT significantly increased cAMP levels in cells expressing the Msep5-HT7S receptor, but showed lower efficacy than 5-HT (Figure 3A,B). We examined the dose–response relationship of 5-HT and Msep5-HT7 receptors in dose concentrations of 5-HT, ranging from 100 pM to 1 mM. The results show that the maximum efficacy of 5-HT against Msep5-HT7L and Msep5-HT7S receptors was equivalent, and displayed dose-dependency and saturability (Figure 3C,D). Half-maximal stimulation (EC_50_) of cAMP production was achieved at 5-HT concentrations of 9.24 × 10^−8^ M (log EC_50_ = −7.03 ± 0.10) and 3.51 × 10^−8^ M (log EC_50_ = −7.46 ± 0.19) in Msep5-HT7L-expressing and Msep5-HT7S-expressing cells, respectively (Table 1). 

The dose–response relationship for synthetic agonists was also measured using the same method, with dose concentrations ranging from 0.1 μM to 1 mM. cAMP responses produced by the highest concentration (1 mM) of synthetic agonists were also consistently poorer than the same concentration of 5-HT induced responses, and the efficacy of synthetic agonists was different between the two receptor isoforms. The highest concentration of αm-5-HT and 5-MT (1 mM) resulted in about 69% activation compared with the maximum amount of cAMP stimulation obtained with the Msep5-HT7L receptor with the same concentration of 5-HT. However, these two agonists resulted in about 35% activation of the maximum amount of cAMP obtained in the Msep5-HT7S receptor. The highest efficacy of 8-OH-DPAT was below 30% of the maximum response of 5-HT in the two isoforms (Figure 3C,D). All tested synthetic agonists exhibited agonistic potency, and the effects were dose-dependent. The EC_50_ values of the synthetic agonists were more than 100-fold higher than those of 5-HT, implying that they were considerably less potent than 5-HT (Table 1). The agonist potency of 8-OH-DPAT against Msep5-HT7L was 2.4-fold higher than against the Msep5-HT7S receptor, while αm-5-HT showed agonist potency against Msep5-HT7S 2.3-fold higher than Msep5-HT7L. The agonist potency of 5-MT was roughly equivalent to the two Msep5-HT7 isoforms (Table 1).

**Figure 4:**
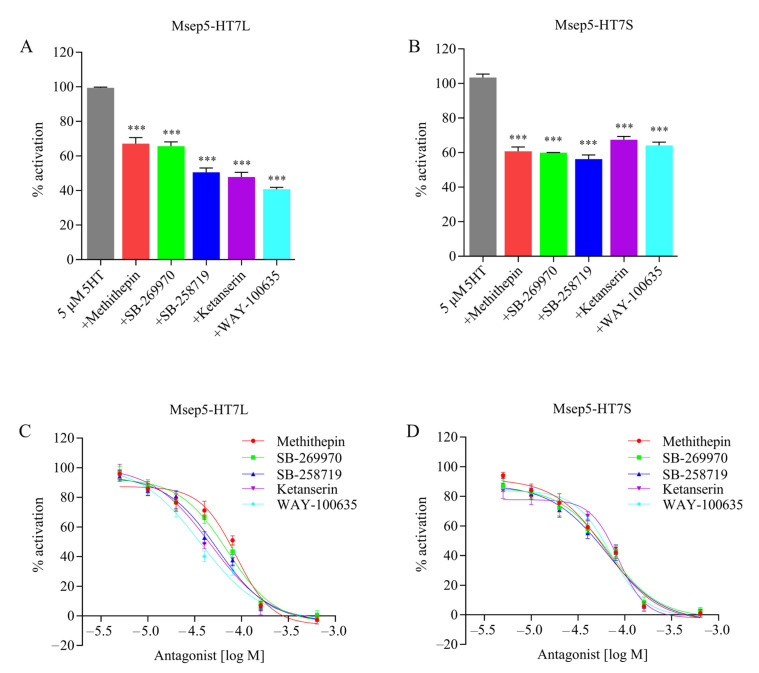
Effects of various potential 5-HT receptor antagonists on HEK293 cells expressing Msep5-HT7L or Msep5-HT7S receptors. For (**A**,**B**), effects of 40 μM putative antagonists, shown as percentage of activation, were achieved with 5 μM of 5-HT (indicated as 100%). Dose-dependent cAMP response to different concentrations of antagonists on HEK293 cells expressing Msep5-HT7L and Msep5-HT7S is shown (**C**,**D**). Asterisks indicate values significantly different from the control value using one-way ANOVA followed by Dunnett’s multiple comparison test (*** *p* < 0.001). cAMP levels of cells treated with 5 μM of 5-HT were defined as 100%; cells treated with only DPBS buffer solution were defined as basal level of 0%. Data are presented as means ± SD from three independent experiments.

Potential synthetic antagonists were tested by simultaneously applying 5 μM of 5-HT and an 8-fold dose of antagonists to stable transfected cell lines expressing Msep5-HT7L or Msep5-HT7L receptors. All of the tested mammalian 5-HT receptor antagonists (methiothepin, ketanserin, WAY-100635, SB-258719, and SB-269970) decreased the 5-HT induced response in Msep5-HT7-expressing cell lines by about 33–60% (Figure 4A,B). The values for the induced receptor response of 5 μM of 5-HT were almost completely blocked by the highest concentration of antagonists (0.64 mM), and the blocking effect of all antagonists on Msep5-HT7 receptor was dose-dependent (Figure 4C,D). The half-maximum inhibitory concentration (IC_50_) values are listed in Table 2. There were no significant differences in the potency or efficacy of the antagonists methithepin, SB-258719, and SB-269970 to inhibit 5-HT-induced cAMP production between Msep5-HT7L and Msep5-HT7S receptors. Meanwhile, higher antagonistic activity of ketanserin and WAY-100635 was measured for the Msep5-HT7L than the Msep5-HT7S receptor based on non-overlap of the 95% fiducial limits.

### 2.3. Expression Profile Analysis of Msep5-HT7 Genes

**Figure 5:**
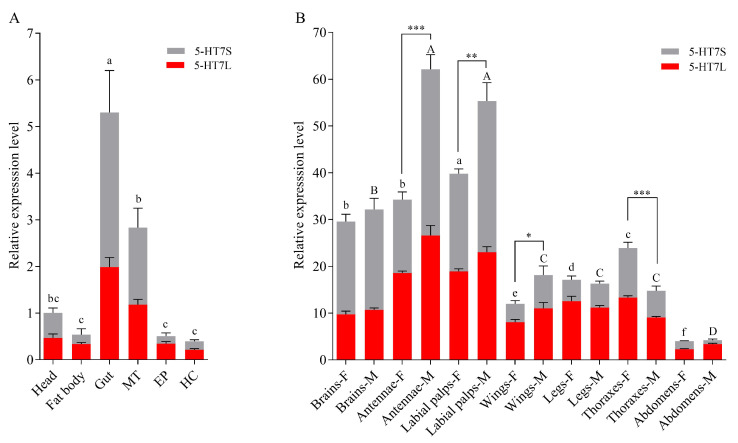
Expression profiles of transcripts encoding Msep5-HT7 in *Mythimna separata*. (**A**,**B**) Relative expression levels of Msep5-HT7S and Msep5-HT7L receptor genes in different tissues of larvae and adults, as evaluated by qPCR. Significant differences in expression in larval tissues using one-way ANOVA and Tukey’s multiple comparison test (*p* < 0.05) are marked above each bar, with lowercase letters in (**A**). Significant differences in expression in male and female tissues using Tukey’s multiple comparison test (*p* < 0.05) are marked on bars with uppercase and lowercase letters. Differences in relative mRNA expression of Msep5-HT7 between sexes were compared by Student’s *t*-test for (**B**); * *p* < 0.05, ** *p* < 0.01, *** *p* < 0.001. MT, malpighian tubules; EP, epidermis; HC, hemocyte. Tissue names with letters F and M indicate female and male, respectively.

*Msep5-HT7* gene has two distinct transcripts, *Msep5-HT7L* and *Msep5-HT7S*, which are generated by alternative splicing. The specific primers that amplify the *Msep5-HT7* gene contain these two transcripts (Figure 1A). The expression profiles of *Msep5-HT7S* and *Msep5-HT7L* in different tissues of larvae and adults were quantified by qPCR (relative expression level of *Msep5-HT7S* = relative expression level of *Msep5-HT7* − relative expression level of *Msep5-HT7L*). The two transcripts were co-expressed in all tested tissues (Figure 5). In fifth instar larvae, *Msep5-HT7* expression was higher in the gut and Malpighian tubules than in other tissues (Figure 5A). It also showed higher expression in the brain, antennae, and labial palps compared to the wing, leg, thorax, and abdomen at the adult stage. This gene had 10-fold higher expression in highly expressed tissue of adult male antennae than in highly expressed tissue of larval gut. In addition, the expression of *Msep5-HT7* was much higher in male than female antennae and labial palps (Figure 5B). We also noticed that the expression ratio of *Msep5-HT7S* in adult wing, leg, thorax, and abdomen tissue was 23–43%. However, this expression ratio in highly expressed adult brain, labial palps, and male antennae was increased to 52–67%. The expression ratio of *Msep5-HT7S* were significantly higher in male than female antennae and labial palps, and was significantly higher in female than male abdomens (*p* < 0.05) (Table 3).

## 3. Discussion

We cloned one *5-HT7* gene in *M. separata* that codes two molecularly distinct transcripts of *5-HT7* by the mechanism of alternative splicing, complying with the typical GU-AG rule. *Msep5-HT7S* differs from *Msep5-HT7L* based on an extra 3′-terminal and 285 nucleotides cleaved off during autocatalytic excision of the intron. The alternative splicing forms of 5-HT7 receptors are widely found in humans, mice, and rats, and alternative splicing occurs extensively at the second and third of three introns of the *5-HT7* gene [2,30,31,32]. Moreover, *5-HT7* receptor genes have been cloned and characterized from *D. melanogaster* [33], *Aedes aegypti* (Linnaeus) (Diptera: Culicidae) [34], *A. mellifera* [27], *C. vicina* [28], *M. sexta* [35], *Tribolium castaneum* (Herbst) (Coleoptera: Tenebrionidae) [36], *P. rapae* [4], and *Plutella xylostella* (Linnaeus) (Lepidoptera: Plutellidae) [37]. Only one type of the receptor gene transcript has been identified in these insects. With the development of genome sequencing technology, many insect genomes have recently been published. This provides an opportunity to analyze the *5-HT7* genomic sequence in different insect species. We checked the coding region of the *5-HT7* gene from *B. mori*, *T. castaneum*, *M. sexta*, *P. rapae*, *P. xylostella*, *Helicoverpa armigera* (Hübner) (Lepidoptera: Noctuidae), and *Trichoplusia ni* (Hübner) (Lepidoptera: Noctuidae) according to the genomic and coding sequence data. Only one intron was found in these insect *5-HT7* genes, which was located in the sequence encoding the putative third intracellular loop. We need to check whether this kind of alternative splicing is a common mechanism in insect *5-HT7* genes in the future. Two Msep5-HT7 receptor isoforms also contain key sequence motifs that are important for ligand binding and signal transduction for biogenic amine receptors, compared with previous reports [27,38,39,40,41,42]. The length of the third intracellular loop varies greatly among insect species (Figure 1C), which may be the reason for the different pharmacological properties of the same agonists and antagonists against the same type of receptors in different insect species.

The 5-HT7 receptor in animals is known to stimulate AC activity, which converts ATP to cAMP, and its coupling with specific G-proteins is brought about by amino acids in close proximity to the plasma membrane of the second and third intracellular loops and the cytoplasmic tail of the receptor proteins [2,27]. This feature is conserved in the two 5-HT7 receptor isoforms of *M. separata*, indicating that these receptor isoforms might have similar signaling pathways. The pharmacological properties of the two receptor isoforms were further explored by testing their downstream signal responses to synthetic agonists and antagonists. The efficacy of 5-HT on the two *M. separata* 5HT7 receptor isoforms was equal, but the potency of 5-HT was nearly three-fold higher against the Msep5-HT7S receptor than the Msep5-HT7L receptor. A similar potency of 5-HT against the 5-HT7 receptor has also been observed in other insects, such as *M. sexta* (EC_50_ = 10.5 nM), *P. rapae* (EC_50_ = 15 nM), *T. castaneum* (EC_50_ = 27.3 nM), and *D. melanogaster* (EC_50_ = 60 nM) [33,35,36]. The synthetic agonists of 8-OH-DPAT, αm-5-HT, and 5-MT were much less efficient and potent than 5-HT in stimulating the two *M. separata* 5-HT7 receptor isoforms. 

Our results are consistent with previous pharmacological studies on 5-HT7 receptors in other insects [4,27,28,32,33,43]. The selective synthetic agonist of 5-carboxamidotryptamine for mammalian 5-HT1 and 5-HT7 receptors has higher potency than 5-HT against mammalian 5-HT7 receptors [44]. However, none of these synthetic agonists seems to be as potent as 5-HT against insect 5-HT7 receptors [4,27,28,36]. For example, the efficacy and potency of αm-5-HT, 5-MT, and 8-OH-DPAT were about 2-fold and more than 190-fold lower, respectively, than 5-HT against the 5-HT7 receptor from *T. castaneum* [36]. All three synthetic agonists have higher efficacy against Msep5-HT7L receptors than Msep5-HT7S receptors. These results indicate that the third intracellular loop of the Msep5-HT7 receptor participates in defining receptor binding sites. 

Five selective or non-selective antagonists of mammalian biogenic amine receptors were effective against *M. separata* 5-HT7 receptor, but their inhibitory potential against 5-HT7 receptors from different insect species were quite different. Methiothepin, a non-selective antagonist against mammalian 5-HT and dopamine receptors, showed about 70- and 480-fold higher inhibitory potency against 5-HT7 receptor from *A. mellifera* (IC_50_ = 0.13 μM) and *P. rapae* (IC_50_ = 0.90 μM), and 125-fold lower inhibitory potency against 5-HT7 receptors from *T. castaneum* (IC_50_ = 7910 μM) than Msep5-HT7S receptors (IC_50_ = 62.86 μM) [4,27,36]. Ketanserin is a specific antagonist against mammalian, *D. melanogaster*, and *C. vicina* 5-HT2A receptors [28,45]. It is also a potent inhibitor of the 5-HT7 receptors from *Caenorhabditis elegans* Maupas (Rhabditida: Rhabditidae), *C. vicina*, and *T. castaneum*, and it showed about 20- to 50-fold higher inhibitory potency against the 5-HT7 receptor from *C. vicina* (IC_50_ = 1.50 μM) and *T. castaneum* (IC_50_ = 1.95 μM) than to Msep5-HT7L (IC_50_ = 46.91 μM) and Msep5-HT7S (IC_50_ = 84.94 μM) [28,36,46]. WAY-100635 is known as a potent inverse agonist against 5-HT1 receptor from *Periplaneta americana* (Linnaeus) (Blattaria: Blattidae) [47], and it was also shown to antagonize the 5-HT1 or 5-HT7 receptor from *M. sexta*, *A. mellifera*, and *T. castaneum* [17,35,39]. It showed similar inhibitory potency against the Msep5-HT7S and 5-HT7 receptors from *T. castaneum* (IC_50_ = 76.8 μM) [36]. SB-269970 and SB-258719 are selective antagonists on the 5-HT7 receptor in mammals [48,49,50]. SB-269970 showed a poor antagonistic effect against the 5-HT7 receptor from *A. mellifera* and *C. vicina*, and moderate inhibitory potency against the 5-HT1 receptor from *T. castaneum* (IC_50_ = 205 μM) [17,27]. However, it showed strong antagonist potency against 5-HT7 receptors from *C. vicina* (IC50 = 9 nM), *P. rapae* (IC_50_ = 1.04 μM), and *T. castaneum* (IC_50_ = 15.82 μM) [4,28,36]. 

Few studies have investigated the effects of SB-258719 on insect 5-HT receptors to date. The antagonistic potency of these two selective 5-HT7 receptor antagonists was statistically equal in terms of the half maximal inhibitory concentration against Msep5-HT7L and Msep5-HT7S. In our study, when the concentration of five antagonists increased 128-fold over 5-HT, they completely blocked 5-HT7 receptor-mediated cAMP production. These antagonists could be used as candidate agents for functional studies. The differences in the potency and efficacy of agonists and antagonists against the 5-HT7 receptor between mammals and insects, and even between different insects, imply that these types of GPCRs could act as potential targets for the development of selective insecticides. 

High transcription levels of *5-HT7* receptor genes were detected in the Malpighian tube, gut, salivary gland, brain, and/or antennae of *A. mellifera*, *G. bimaculatus*, *M. sexta*, *A. aegypti*, *C. vicina*, *T. castaneum*, and *Polyrhachis vicina* Roger (Hymenoptera: Formicidae) [27,28,34,35,36,51,52]. Our results also reveal that the *5-HT7* receptor genes are highly expressed in larval Malpighian tubes and guts, indicating that the 5-HT7 receptor potentially has a role in digestion and metabolism. Yang et al. [52] reported that the *5-HT7* receptor’s mRNA and protein expression were slightly higher in the pupal than the egg and larval stages, and they deduced that this receptor may be involved in modulating adult formation and caste differentiation in *P. vicina*. Moreover, the *5-HT7* gene showed strong expression in the brains of adult *D. melanogaster*, and it mediated normal courtship and mating behaviors [26]. We also noticed that higher transcript numbers of *5-HT7* were found in adult males than females of *P. vicina* and *P. xylostella*, as well as in our detected male *M. separata* antennae and labial palps. In addition, 5-HT-immunoreactive neurons were found to be distributed in the antennal lobe of *M. sexta*. It was then confirmed that 5-HT changes K+ conductance in the cells and increases the excitability and responsiveness of central olfactory neurons to sex pheromones [14,53], although it is unclear which type of 5-HT receptor mediates intracellular signal transduction. High expression of *5-HT7* receptor genes in male antennae and labial palps should receive careful consideration, as it may contribute to modulating sensitivity and specificity to carbon dioxide and plant volatiles, perception of sex pheromones, and regulating courtship and mating behaviors. High expression of *Msep5-HT7S* in male antennae and labial palps could be attributed to its higher sensitivity to endogenous 5-HT than Msep5-HT7L receptor, and organisms that express *Msep5-HT7S* transcript can improve energy efficiency. 

The oriental armyworm *M. separata* is a typical seasonal, long-distance migratory polyphagous pest that can attack nearly 100 families of more than 300 kinds of food and industrial crops, resulting in serious yield losses in China and other parts of Asia and Oceania [54,55,56]. The use of chemical insecticides is the main strategy to control this pest. However, the long-term use of insecticides leads to the development of resistance to traditional insecticides [57]. Thus, there is an urgent need to develop novel insecticides. The potential of 5-HT receptors as insecticide targets has frequently been considered, and some progress has been made toward insecticide discovery through the study of their pharmacological characterization and the design of chemical compounds, with the 5-HT receptor agonist as the lead compound [21,22]. With the present study, detailed knowledge of the pharmacological properties of the Msep5-HT7 receptor and established cell lines that stably express this receptor should facilitate designing, synthesizing, and screening novel insecticides. 

## 4. Materials and Methods

### 4.1. Insects Rearing, Tissue Collection and Reagents

The larvae of *M. separata* were reared on an artificial diet in the climate incubator and maintained under a photoperiod of 14:10 h (light:dark) at 27 °C and 75% relative humidity [58]. Adult moths were provided with 10 % (weight by volume) sucrose solution. Various tissues from fifth instar larvae, including heads, epidermis, fat bodies, Malpighian tubules, and guts, were immediately dissected, and hemocytes were collected by cutting the thoracic legs. Various tissues from three day-old unmated female or male moths including brains, thoraxes, abdomens, antennae, labial palps, wings, and legs were collected, and all samples were used for RNA extraction. The reagents used in the pharmacological ligands, including serotonin hydrochloride (5-HT), (±)-octopamine hydrochloride (OA), dopamine hydrochloride (DA), tyramine hydrochloride (TA), (±)-8-hydroxy-2-(dipropylamino) tetralin hydrobromide (8-OH-DPAT), forskolin, 5-methoxytryptamine (5-MT), methiothepin mesylate, α-methylserotonin maleate (αm-5-HT), WAY-100635, SB-258719, and SB-269970 hydrochloride were purchased from Sigma-Aldrich (St Louis, MO, USA). Ketanserin was purchased from MedChemExpress (Monmouth Junction, NJ, USA).

### 4.2. Cloning of M. separata 5-HT7 Receptor Gene

Genomic DNA and total RNA were extracted from adult *M. separata* using an EasyPure Genomic DNA Kit (Transgen Biotech, Beijing, China) and TRIzol reagent (Invitrogen, Carlsbad, CA, USA), respectively. Genomic DNA and single-stranded cDNA synthesized from the RNA using the Fast King gDNA Dispelling RT SuperMix (TianGen, Beijing, China) were used as the template for PCRs to amplify the genomic DNA sequences and open reading frame (ORF) of *Msep5-HT7*, respectively. Full-length sequences of putative *Msep5-HT7* were obtained from our previous brain transcriptomes of *M. separata* [59]. The gene-specific primers (Table 4) used in our studies were designed by Primer Premier 5.0 software. The amplified DNA fragments were cloned into the pMD19-T Simple Vector (Takara, Dalian, China). Positive clones were identified and sequenced by Sangon Biotech (Shanghai, China). The obtained cDNA sequences were submitted to NCBI as *Msep5-HT7L* (GenBank Acc. OM025087) and *Msep5-HT7S* (GenBank Acc. OM025088).

### 4.3. Multiple Sequence and Phylogenetic Analysis

Alignments of 5-HT7 receptor amino acid sequences were performed by MAFFT online, and the results were displayed by ESPRIPT [60]. The transmembrane segments were predicted by TMHMM 2.0 online. To identify the potential orthology of the identified *M. separata* 5-HT7 receptor, the phylogenetic trees of 5-HT receptors from other insects were constructed using MEGA 6.0 software. The maximum-likelihood method was used with a bootstrap analysis of 1000 replicates, based on the JTT matrix-based model [61]. The *Drosophila melanogaster* FMRF amide receptor was used as outgroup. The amino acid sequences used in the phylogenetic analyses are listed in Supplementary Materials.

### 4.4. Construction of Expression Plasmids

The construction of expression plasmids of the *Msep5-HT7L* and *Msep5-HT7S* cDNA containing the Kozak consensus sequence (GCCACC) [62] were obtained by PCR amplification with specific primers (Table 4). The DNA fragments were double digested with *KpnI* and *XhoI* (Sangon Biotech, Shanghai, China), and after purification, they were cloned into a pcDNA3.0 vector (Invitrogen, Carlsbad, CA, USA). DNA sequencing was used to confirm the correct recombinant plasmid.

### 4.5. Cell Culture, Transfection, and Creation of Stable Cell Lines

HEK-293 cells were cultured in D-MEM (Gibco BRL, Gaithersburg, MD, USA) supplemented with 10% FBS (Gibco BRL, Gaithersburg, MD, USA) at 37 °C and 5% CO_2_. Four microgram recombinant plasmids with 10 μL of lipofectamine 2000 (Invitrogen, Carlsbad, CA, USA) were introduced into exponentially growing HEK 293 cells. The 0.8 mg/mL antibiotic G418 (Sigma, St. Louis, MO, USA) was added to the medium to select for cells that were stably expressed the Msep5-HT7 receptors. After fourteen days of G418 selection, three G418-resistant colonies were trypsinized in cloning cylinders and transferred to 12-well plastic plates for expansion [20].

### 4.6. cAMP Assays

Intracellular cAMP concentrations were measured as previously described [4,63,64]. Briefly, when cells were cultured for 2 days to the concentration of 1 × 10^6^ cells/mL, culture media were removed and the cells were washed two times with cold PBS (pH 7.4), then the cells were preincubated in 450 μL of Dulbecco’s phosphate-buffered solution (DPBS) containing 100 μM phosphodiesterase inhibitor 3-isobutyl-1-methylxanthine (IBMX) for 20 min at room temperature. After the preincubation, 50 μL of DPBS, containing various concentrations of reagents, was added into the stable transfected cells for incubation for 30 min at 37 °C. The cells were washed three times with cold PBS (pH 7.4) and then lysed in 250 μL of cold cell lysis buffer by re-freezing. The cell debris was removed by centrifugation at 600× *g* for 10 min at 4 °C. The amount of intracellular cAMP extracted from harvested supernatant was determined using an ELISA reagent (R&D Systems, Minneapolis, MN, USA). Each measurement was performed in duplicate, and three independent assays were carried out for each reagent and concentration tested.

### 4.7. Expression Profiles Analysis by qPCR

Real-time PCR was used to confirm the expression profiles of the *Msep5-HT7* gene in larvae and various adult tissues. cDNA was synthesized using total RNA that was extracted from above collected samples with the FastKing gDNA Dispelling RT SuperMix (TianGen, Beijing, China). The specific qPCR primers used to amplify *Msep5-HT7* and the reference genes are listed in Table 4. The reference genes *β-actin* (GenBank Acc. GQ856238.1) and glyceraldehyde-3-phosphatedehydrogenase (*gapdh*) (GenBank Acc. HM055756.1) were used to normalize *Msep5-HT7* gene expression. Reactions for each sample (20 μL) consisted of 10 μL of 2 × SuperReal PreMix Plus (TianGen, Beijing, China), 7.6 μL of sterilized H_2_O, 0.5 μL of each primer (10 μM), 0.4 μL of Rox reference dye, and 1 μL of cDNA. qPCR was carried out with an initial denaturation at 95 °C for 3 min, followed by 40 cycles at 95 °C for 15 s and 60 °C for 30 s. The qPCR reaction of each sample was performed in three technical replicates and three biological replicates. Then, we used a relative quantitation method (2^−ΔΔCT^) [65] to evaluate quantitative variation. Transcript amounts were standardized to 1 with the sample from the heads of fifth-instar larvae.

### 4.8. Statistical Analysis

GraphPad Prism 7.0 software was used for curve fitting and statistical analysis. We used one-way analysis of variance (ANOVA) with Tukey’s multiple comparison test (*p* < 0.05) to analyze the expression differences of the *Msep5-HT7* gene among different larvae and adult tissues. Then, we used Student’s *t*-test to test for statistically significant differences in *Msep5-HT7* expression levels and expression ratio of *5-HT7S* between the same tissues from male and female adults, respectively (*p* < 0.05).

## Figures and Tables

**Table 1:** EC_50_ values of agonists to activation of two Msep5-HT7 receptor isoforms in HEK-293 cells.

Agonist	5-HT7L		5-HT7S	
	EC_50_ (μM)	logEC_50_ (Mean ± SEM)	EC_50_ (μM)	logEC_50_ (Mean ± SEM)
5-HT	0.092	−7.03 ± 0.10	0.035	−7.46 ± 0.19
5-MT	20.91	−4.68 ± 0.07	19.42	−4.71 ± 0.22
αm-5-HT	13.89	−4.86 ± 0.04	5.90	−5.23 ± 0.29
8-OH-DPAT	10.06	−5.00 ± 0.11	24.82	−4.61 ± 0.30

**Table 2:** IC_50_ values of antagonists against two Msep5-HT7 receptor isoforms activation on HEK-293 cells.

Antagonist	5-HT7L		5-HT7S	
	IC_50_ (μM)	logIC_50_ (Mean ± SEM)	IC_50_ (μM)	logIC_50_ (Mean ± SEM)
Methithepin	87.21	−4.06 ± 0.03	62.86	−4.20 ± 0.03
Ketanserin	46.91	−4.33 ± 0.04	84.94	−4.07 ± 0.02
WAY-100635	36.15	−4.44 ± 0.04	70.33	−4.15 ± 0.02
SB-258719	55.26	−4.26 ± 0.03	64.04	−4.19 ± 0.04
SB-269970	70.36	−4.15 ± 0.03	67.78	−4.17 ± 0.03

**Table 3:** Expression ratio of *5-HT7S* in adult tissues of *Mythima separata*.

Tissue	Sex	Expression Ratio (Mean ± SEM%) ^a^	*p* ^b^
Brain	F	66.39 ± 0.60	0.71
	M	67.19 ± 1.95	
Antennae	F	44.87 ± 0.94	0.02
	M	56.33 ± 2.64	
Labial palp	F	52.42 ± 1.01	0.04
	M	57.95 ± 1.61	
Thorax	F	42.86 ± 4.95	0.35
	M	36.81 ± 2.74	
Wing	F	34.96 ± 3.88	0.47
	M	38.48 ± 2.13	
Leg	F	33.48 ± 4.49	0.74
	M	31.06 ± 5.16	
Abdomen	F	43.46 ± 1.46	0.01
	M	22.87 ± 3.82	

^a^ Expression ratio = relative expression level of *Msep5-HT7S*/relative expression level of *Msep5-HT7* × 100%. ^b^ Probability of difference in expression ratio between female and male based on Student’s *t*-test.

**Table 4:** Primers used in this study.

Primer Names	Primer Sequence (5′-3′)
For complete cDNA and gDNA cloning	
5-HT7-comp-F	ATGGCGACTCAAAGTCATAACTCCCACTGTTCAC
5-HT7-comp-R	TAGAAAACTTTCCGAAGCCCGCGTCTCCTCTC
For RT-PCR and real-time PCR	
5-HT7-RTF	CCTCCTCCGAGAGGATCTACTGC
5-HT7-RTR	TTGGCTAGTTGGAACCGCAGC
5-HT7-qPCR-F	GGACCTTCTTAGACGACAACTCCAC
5-HT7-qPCR-R	TCGCTTACTGCCAGCGACACTATC
5-HT7L-qPCR-F	CGAACGAGTCCCAGTGTCCTATCT
5-HT7L-qPCR-R	GATCGGATTCTGTTCGTCGTATTCT
β-actin-qPCR-F	AACTTCCCGACGGTCAAGTCAT
β-actin-qPCR-R	TGTTGGCGTACAAGTCCTTACG
GAPDH-qPCR-F	ATGTTCGTGTGCGGAGTCAAC
GAPDH-qPCR-R	TCTTCTGGGTAGCGGTGGTAG
For eukaryotic expression	
Msep5-HT7-KpnⅠ-F	GG*GGTACC***GCCACC**ATGGCGACTCAAAGTCATAACTCC
Msep5-HT7-XhoⅠ-R	CCG*CTCGAG*TCATAGAAAACTTTCCGAAGCCCGCGTCTCCT

## Data Availability

Data are contained within the article or Supplementary Material.

## Supplementary Materials

The following supporting information can be downloaded at: https://www.mdpi.com/article/10.3390/ijms24010655/s1.

## Abbreviations

5-HT	serotonin
OA	octopamine
TA	tyramine
DA	dopamine
8-OH-DPAT	8-Hydroxy-DPAT
5-MT	5-methoxytryptamine
αm-5-HT	α-methylserotonin
TM	transmembrane
GPCRs	G protein-coupled receptors
HEK 293	Human Embryonic Kidney 293
cAMP	cyclic adenosine monophosphate
Ac	adenylyl cyclase
IBMX	3-isobutyl-1-methylxanthine
PBS	phosphate buffered solution
DPBS	Dulbecco’s phosphate buffered solution
FBS	fetal bovine serum
D-MEM	Dulbecco’s Modified Eagle Medium
RT-PCR	reverse transcription-polymerase chain reaction
qPCR	quantitative real-time polymerase chain reaction
NCBI	National Center for Biotechnology Information
